# The association between occupational lead exposure and serum levels of vitamin D3 and a bone turnover biomarker in smelter workers

**DOI:** 10.1007/s00420-025-02125-y

**Published:** 2025-03-08

**Authors:** Rateba Said Mohammed, Basma Hussein Mourad

**Affiliations:** Department of Occupational and Environmental Medicine, Faculty of Medicine, Cairo University, Cairo, Egypt

**Keywords:** Chronic occupational pb exposure, Vitamin D3, Biomarker of bone turnover, CTX-1, Smelters workers

## Abstract

**Objective:**

Bone tissue is the chief target for lead (Pb) in chronic exposure. This study aimed to demonstrate the relation between the blood lead levels (BLL) and serum levels of 1,25 dihydroxy cholecalciferol (vitamin D3) and type I collagen cross-linked C-telopeptide (CTX-1) as a biomarker of bone turnover among some Egyptian workers occupationally exposed to Pb in the smelting process. The study also targeted to identify any clinical manifestations indicative of skeletal system affection and their association with the performed investigations.

**Methods:**

A total of 48 smelter workers and 48 administrative controls participated in the study. All subjects underwent comprehensive medical and occupational history taking and detailed clinical examinations, with a particular focus on symptoms indicative of skeletal system involvement. These symptoms included generalized bony aches, low back pain, joint pain, restricted joint movement, a history of fractures from minor trauma, and delayed fracture healing. BLL, as well as serum levels of vitamin D3 and CTX-1, were measured in all participants.

**Results:**

Smelter workers exhibited significantly higher prevalence of bony aches, low back pain, joint pain, and past fractures from minor trauma compared to controls. The BLL and serum CTX-1 levels were significantly elevated in the exposed group, while serum vitamin D3 levels were notably lower. Logistic regression analysis revealed that BLL significantly predicted bony aches and low back pain. Additionally, serum vitamin D3 and CTX-1 levels were significant predictors of low back pain and joint pain, respectively, among exposed workers. The measured parameters were significantly correlated with one another and with the duration of employment in the exposed group.

**Conclusion:**

Significant associations between manifestations of skeletal system affection, BLL, and serum levels of vitamin D3 and CTX-1 were detected among smelter workers with chronic occupational exposure to Pb.

## Introduction

When non-ferrous metals are smelted at high temperatures, some metals can gasify and become suspended in the air. Lead (Pb) is one of the most commonly encountered heavy metals during the smelting process. As a semi-volatile heavy metal, Pb tends to evaporate from ore particles at high temperatures during smelting, remaining in a gaseous state or condensing into fine particulate matter (PM) within cooling flue gas (Zhang et al. 2022). This often results in elevated Pb levels in the industrial environment. Occupational exposure to Pb typically occurs through inhalation of Pb dust or fumes, with ingestion and dermal absorption playing lesser roles (CDC 2024a). Once absorbed, Pb binds to blood proteins and is distributed to various soft and hard tissues. The bone matrix is a primary target for Pb, as it can substitute for other divalent cations, including Ca²⁺, Mg²⁺, and Fe²⁺ (Rodríguez and Mandalunis 2018).

Bone tissue consists of several cell types, including osteocytes, osteoblasts, osteoclasts, and bone lining cells, which are embedded within a calcified extracellular matrix. The bone matrix is composed of minerals (50–70%), organic matter (20–40%), and water (5–10%). Type I collagen, synthesized by osteoblasts and deposited in layers within the mature (lamellar) bone, constitutes approximately 90% of the organic component of the extracellular matrix (Schlesinger et al. 2020). Osteoclastic activity has been linked to bone resorption, characterized by the release of type I collagen cross-linked C-telopeptide (CTX-1), a degradation product of collagen (Ying et al. 2023).

Extensive laboratory and clinical studies have shown that long-term Pb exposure adversely affects bone tissue function through two primary mechanisms. First, Pb directly impacts bone tissue, reducing bone mass and density by inhibiting osteoblast and chondrocyte activity, inducing cytotoxicity, and triggering apoptosis in mesenchymal stem cells (Sharifi et al. 2011; Akbal et al. 2014). Second, Pb exhibits a high affinity for thiol groups in the active sites of various enzymes, including kidney 1-α-hydroxylase, which is crucial for synthesizing 1,25-dihydroxycholecalciferol (vitamin D3). The inhibition of this enzyme by Pb, disrupts calcium and phosphorus metabolism, resulting in decreased intestinal absorption, hypocalcemia, and hypophosphatemia (Dongre et al. 2013).

Decreased bone mineral density, along with Pb deposition in the cement lines of bone and increased collagen resorption, leads to delayed cartilage formation, a heightened risk of bone fractures, osteoporosis, and impaired fracture healing (Ravibabu et al. 2020).

Taking the aforementioned into consideration, the current study was designed to investigate the association between blood Pb levels and serum levels of vitamin D3 and CTX-1 as a biomarker of bone turnover in a subset of Egyptian workers occupationally exposed to Pb in a non-ferrous metal smelter. Additionally, the study aimed to identify clinical symptoms or complaints indicative of skeletal system involvement and their correlation with laboratory findings.

## Methods and materials

### Study design, setting, and population

This cross-sectional comparative study was conducted at a non-ferrous smelter in Helwan, Greater Cairo, Egypt, between May and June 2024. Workers in this facility are exposed to lead (Pb) during various high-risk activities involved in processing non-ferrous metals such as aluminum and copper. Their tasks include loading metal ores or concentrates into furnaces, which release Pb fumes and dust into the air during the smelting process.

Additionally, workers are engaged in refining molten metals, where chemical treatments and high-temperature operations further vaporize Pb compounds, increasing the risk of inhalation exposure. Maintenance activities, such as cleaning furnaces, repairing equipment, and managing slag, often disturb accumulated Pb dust, leading to significant exposure through both inhalation and dermal contact. Furthermore, tasks such as sampling or quality testing of molten metal frequently expose workers to direct Pb fumes.

Based on the sample size calculation, the study included 48 smelter workers with chronic occupational exposure to Pb and a control group of 48 administrative personnel who were not exposed to Pb. To qualify for the study, each exposed worker had to have been actively involved in the production process within the industrial setting for a minimum of two years. Notably, all exposed workers reported proper use of personal protective equipment (PPE). The PPE utilized included N95 air-purifying respirators, safety goggles, face shields, heat-resistant and flame-retardant suits, overalls, gloves, and boots. Additionally, the local exhaust ventilation system appeared to function effectively.

Environmental monitoring of heavy metals, including lead (Pb), aluminum (Al), and copper (Cu), is conducted annually within the smelter to ensure compliance with national safety standards. Data from these monitoring efforts consistently show that the concentrations of these metals in the workplace air and surrounding environment remain within the permissible limits set by national regulatory bodies. The authors reviewed the results of this monitoring in collaboration with the smelter administration; however, the data were not made available for inclusion in this study. According to the Egyptian Environmental Affairs Agency (EEAA), the threshold limit value (TLV) for Pb in workplace air is set at 0.05 mg/m³ (50 µg/m³), averaged over an 8-hour workday. For Al and Cu, the EEAA has established TLVs of 5 mg/m³ and 1 mg/m³, respectively, for respirable dust fractions (EEAA 1995).

*Sample size calculation*: The Power and Sample Size Calculator tool (version 3.0.43) was used to determine the sample size. The calculation was based on the following inputs: a significance level of 0.05, a mean difference between groups (g1 and g2) of 0.58, and a total sample size of 96 participants, with 48 individuals in each group (Yadav et al. 2018).

Participants with endocrine or metabolic disorders affecting bone tissue, or those using medications known to influence calcium-phosphate metabolism and bone turnover, were excluded from the study.

To form the control group, 48 matched individuals without a history of occupational Pb exposure were selected from the administrative department of the investigated smelter. After applying the exclusion criteria, control participants were chosen to match the exposed group in terms of sex, age, socioeconomic status, and specific medically significant behaviours, such as smoking.

The Research Ethics Committee of the Faculty of Medicine, Cairo University, Egypt, approved the study (approval number: N-467-2023). The Chief Executive Officer (CEO) of the smelter also presented formal consent for the study.

After receiving a detailed explanation of the study’s purpose, each participant voluntarily provided written informed consent to participate. Strict confidentiality and adherence to good clinical practice principles were maintained throughout the processes of sample collection, testing, coding, and recording of results.

### Data collection

A team of experienced occupational doctors conducted interviews with the participants to gather personal and occupational information. The medical history and clinical examination focused on any complaints or signs indicating skeletal system involvement, including generalized bone aches, back pain, joint pain, limited joint movement, a history of fractures from minor impact or low-energy activities, and delayed healing of previous fractures.

### Blood sample collection and laboratory investigations

Five millilitres of whole blood were drawn from each participant at the end of the work shift: 2 mL in heparinized tubes and 3 mL in plain tubes. The 2-mL sample was used to estimate blood lead levels (BLL). The 3-mL sample was centrifuged at 4 °C and 3000 RPM for 10 min to extract serum for measuring serum vitamin D3 and CTX-1. Laboratory assays were conducted at the Clinical and Chemical Pathology Department Laboratory, Faculty of Medicine, Cairo University, Egypt.

#### Measurement of blood lead levels

Until analysis, the 2-mL blood sample was kept at -20 °C. The sample was digested with 2 mL of nitric acid and 0.2 mL of hydrogen peroxide using ETHOS-D (Milestone Microwave Laboratory Systems, Italy) while keeping power, temperature, and process duration constant. Using distilled water, the digested samples were diluted to a volume of 5 mL and then centrifuged. Using an atomic absorption spectrophotometer (GBC Avanta P, Australia), the concentration of Pb was determined. For the sample with the lowest concentration, a standard solution containing 20 µg/dL of lead was generated using a stock solution. After three repetitions, the study revealed 100% recovery with % relative standard deviation (RSD) at < 0.5 (Kalahasthi et al. 2016).

#### Measurement of serum vitamin D3 and CTX-1

The enzyme-linked immunosorbent assay (ELISA) was used to determine the amounts of serum vitamin D3 and CTX-1 (BT LAB Bioassay Technology Laboratory, Shanghai, China). Thermo Scientific Multiskan EX-reader (USA) was used to detect the absorbance of samples and standard specimens at 450 nm. The unknown sample’s concentration was determined through the use of a standard curve and linear regression. For vitamin D3 and CTX-1, the range of the technique was 7–500 pg/mL and 7–1500 ng/mL, respectively. For vitamin D3 and CTX-1, the method’s sensitivity was 3.14 pg/mL and 4.21 ng/mL, respectively (Ali et al. 2024).

### Statistical analysis

The statistical software for the social sciences (SPSS) version 28 (IBM Corp., Armonk, NY, USA) was used to code and input the data. For quantitative variables, the mean, standard deviation, median, minimum, and maximum were used to summarize the data; for categorical variables, the frequencies (number of cases) and relative frequencies (percentages) were used. For normally distributed quantitative variables, group comparisons were carried out using the unpaired t-test; for non-normally distributed quantitative variables, the non-parametric Mann-Whitney test was employed (Chan 2003a). The normally distributed variables were the age, BMI, and levels of BLL, vitamin D3 and CTX-1 of the studied participants. The Chi-square (χ2) test was used to compare categorical data. When the predicted frequency is less than five, the Exact test was utilized in its place. A 95% confidence interval for the odds ratio (OR) was computed (Chan 2003b). The Spearman correlation coefficient was used to perform correlations between quantitative variables (Chan 2003c). Logistic regression was done to detect independent predictors of the clinical manifestations of skeletal system affection (Chan 2004a). For identifying independent predictors of vitamin D3 and CTX-1 levels, linear regression analysis was used (Chan 2004b). The significance level of 0.05 for a P-value was deemed statistically significant.

## Results

The general demographic details of the exposed and comparison groups are presented in Table 1. There were no statistically significant differences between the exposed smelter workers and the administrative controls in terms of age, body mass index (BMI), smoking index, or duration of employment. The age of the smelter workers ranged from 25 to 58 years, and their employment duration ranged from 5 to 25 years.

**Table 1:** Comparison between the exposed and control groups as regards their general characteristics, BLL, and their serum levels of vitamin D3 and CTX-1

	Exposed group (*n* = 48)					Control group (*n* = 48)					*P* value
	Mean	SD	Median	Minimum	Maximum	Mean	SD	Median	Minimum	Maximum	
Age (years)	37.29	10.07	34	25	58	38.71	10.34	36	25	57	0.498
BMI	28.72	5.69	28.45	19.5	40	29.4	4.32	29.4	20.7	39.7	0.512
Smoking index	97.92	162.41	0	0	700	91.67	155.17	0	0	500	0.479
Duration of employment (years)	11.04	4.81	9	5	25	12.19	6.66	9	4	32	0.837
BLL (µg/dl)	11.86	3.56	11.9	3.60	20	4.30	1.40	4	2	7.2	< 0.001*
Vitamin D3 (pg/mL)	29.42	12.03	31.6	8.3	72.64	48.23	21.22	44.05	8.1	122	< 0.001*
CTX-1 (ng/mL)	725.73	233.79	704.5	139.1	1177	393.15	188.63	364.1	65.3	813.9	< 0.001*

SD = Standard deviation; BMI = Body Mass Index; Smoking index = The number of cigarettes smoked per day multiplied by the number of years of smoking; BLL = Blood Lead Level; CTX-1 = Type I collagen cross-linked C-telopeptide

*p-value < 0.05 denotes statistical significance

In contrast, the mean blood lead level (BLL) in the exposed group (11.86 ± 3.56 µg/dL) was significantly higher than that in the comparison group (4.30 ± 1.40 µg/dL) (*p*-value < 0.001). The mean serum level of vitamin D3 was significantly lower in the exposed smelter workers (29.42 ± 12.03 pg/mL) compared with the administrative controls (48.23 ± 21.22 pg/mL). Additionally, the mean serum level of the bone turnover biomarker CTX-1 was significantly elevated in the exposed group (725.73 ± 233.79 ng/mL) compared with the control group (393.15 ± 188.63 ng/mL) (Table 1).

Medical history and clinical examination revealed that symptoms indicating skeletal system involvement were significantly more common (*p* < 0.05) among exposed workers compared to the control group (Table 2). Recurrent bony aches were reported by 47.9% of exposed workers (*n* = 23), with an odds ratio (OR) of 6.44 (95% CI: 2.309–17.964). Additionally, 41.7% (*n* = 20) reported prolonged low back pain (OR: 3.571, 95% CI: 1.379–9.249), and 35.4% (*n* = 17) experienced pain in multiple joints (OR: 4.716, 95% CI: 1.572–14.152). Past fractures after minor trauma were reported by 27.1% (*n* = 13) of workers, showing a statistically significant difference compared to the control group (OR: 5.571, 95% CI: 1.472–21.083). In the administrative control group, the prevalence of bony aches, low back pain, joint pain, and past fractures was 12.5%, 16.7%, 10.4%, and 6.3%, respectively. Additionally, 10 exposed smelter workers reported limited joint movement, and 5 workers reported delayed healing of past fractures. However, the prevalence of these two symptoms did not differ significantly from the control group.

**Table 2:** The frequency distribution of the exposed and control participants with some clinical manifestations of skeletal system affection

Manifestation	Exposed group (*n* = 48)		Control group (*n* = 48)		X^2^	*P* value	OR	95% CI	
	No.	%	No.	%				Lower	Upper
Bony aches	23	47.9%	6	12.5%	14.279	< 0.001*	6.440	2.309	17.964
Low back pain	20	41.7%	8	16.7%	7.261	0.007*	3.571	1.379	9.249
Joint pain	17	35.4%	5	10.4%	8.491	0.004*	4.716	1.572	14.152
History of past fracture after minor trauma	13	27.1%	3	6.3%	7.500	0.006*	5.571	1.472	21.083
Limited joint movement	10	20.8%	5	10.4%	1.975	0.160	2.263	0.710	7.211
History of delayed healing of fracture	5	10.4%	1	2.1%	2.844	0.204	5.465	0.614	48.662

*p-value < 0.05 denotes statistical significance

Logistic regression analysis was performed to predict the clinical manifestations of skeletal system involvement among exposed smelter workers using blood lead level (BLL), vitamin D3, and CTX-1 serum levels as independent variables (Table 3). The results showed that BLL was a significant predictor of bony aches and low back pain, with an odds ratio (OR) greater than 1. Also, serum vitamin D3 levels significantly predicted low back pain, while serum CTX-1 levels were significant predictors of joint pain, both with ORs greater than 1.

**Table 3:** Logistic regression analysis to predict the clinical manifestations of skeletal system affection using BLL, and serum levels of vitamin D3 and CTX-1 as independent variables among the exposed group

		P value	OR	95% C.I.	
				Lower	Upper
Bony aches	BLL	0.041*	1.014	1.000	1.029
	Vitamin D3	0.495	1.004	0.992	1.017
	CTX-1	0.332	0.677	0.308	1.488
Low back pain	BLL	0.036*	1.066	1.032	1.098
	Vitamin D3	0.028*	1.074	1.055	1.089
	CTX-1	0.312	0.629	0.256	1.545
Joint pain	BLL	0.816	1.025	0.834	1.260
	Vitamin D3	0.386	1.006	0.993	1.020
	CTX-1	0.042*	1.034	1.017	1.057
History of past fracture after minor trauma	BLL	0.920	0.983	0.697	1.385
	Vitamin D3	0.158	1.011	0.996	1.027
	CTX-1	0.298	0.591	0.219	1.592
Limited joint movement	BLL	0.816	1.025	0.834	1.260
	Vitamin D3	0.386	1.006	0.993	1.020
	CTX-1	0.482	0.732	0.307	1.747
History of delayed healing of fracture	BLL	0.920	0.983	0.697	1.385
	Vitamin D3	0.158	1.011	0.996	1.027
	CTX-1	0.298	0.591	0.219	1.592

BLL = Blood Lead Level; CTX-1 = Type I collagen cross linked C-telopeptide

*p-value < 0.05 denotes statistical significance

To identify significant independent predictors for serum vitamin D3 and CTX-1 levels among the exposed workers, linear regression analyses were conducted. The findings (Table 4) revealed that BLL was a statistically significant independent predictor of serum vitamin D3 and CTX-1 levels in the exposed participants. Moreover, the duration of employment among the exposed workers was also identified as a statistically significant independent predictor of serum CTX-1 levels.

**Table 4:** Linear regression analyses to identify significant independent predictors for the serum levels of vitamin D3 and CTX-1 among the exposed smelter workers

Model			Unstandardized Coefficients		Standardized Coefficients	t	P value	95.0% Confidence Interval for B	
			B	Std. Error	Beta			Lower Bound	Upper Bound
Vitamin D3	(Constant)		66.920	1.631		41.026	**< 0.001**	63.635	70.206
	BLL		-1.445	0.556	-0.428	-2.601	0.013*	-2.564	-0.326
CTX-1		(Constant)	444.821	140.325		3.170	**0.003**	162.014	727.628
		BLL	38.893	6.976	0.592	5.575	< 0.001*	24.833	52.953
		Duration of employment	3.186	1.541	0.065	2.068	0.045*	0.080	6.291

BLL = Blood Lead Level; CTX-1 = Type I collagen cross linked C-telopeptide

*p-value < 0.05 denotes statistical significance

**Fig. 1:**
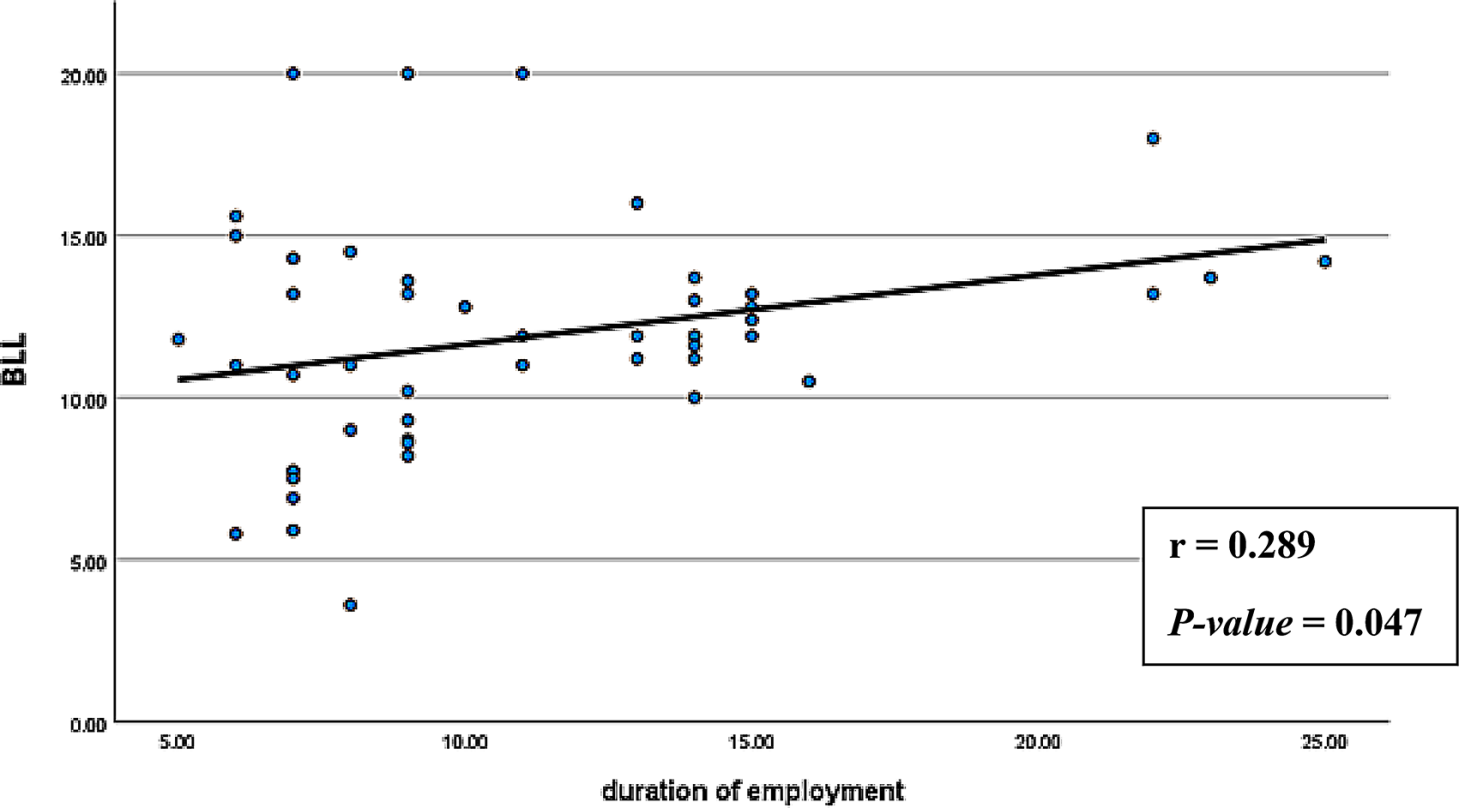
Correlation between the duration (years) of employment and the blood lead levels (BLL) among the exposed group (*r* = 0.289, *P*-value = 0.047)

**Fig. 2:**
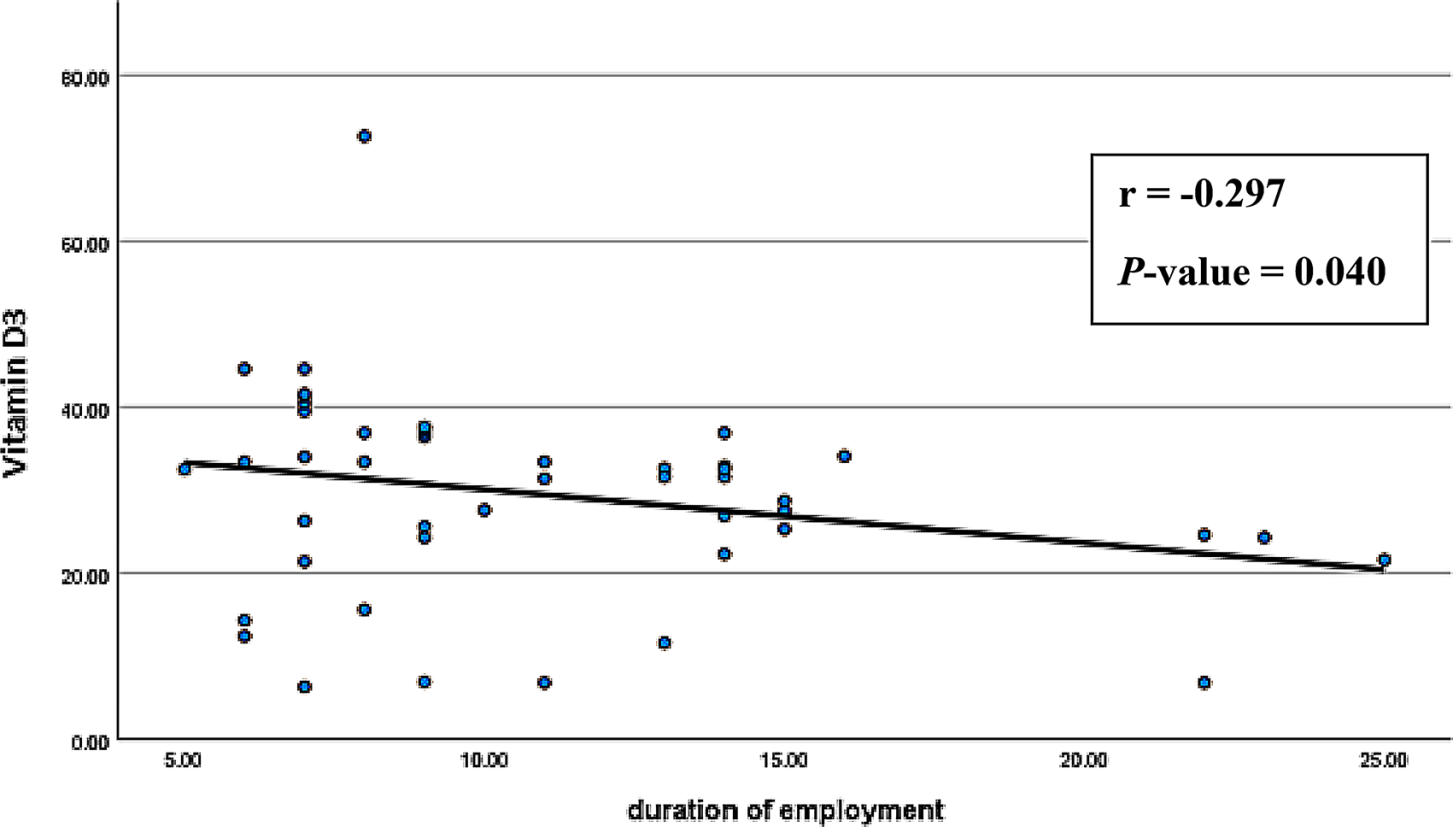
Correlation between the duration (years) of employment and the serum levels of 1,25 dihydroxy-cholecalcifirol (vitamin D3) among the exposed group (*r* = -0.297, *P*-value = 0.040)

**Fig. 3:**
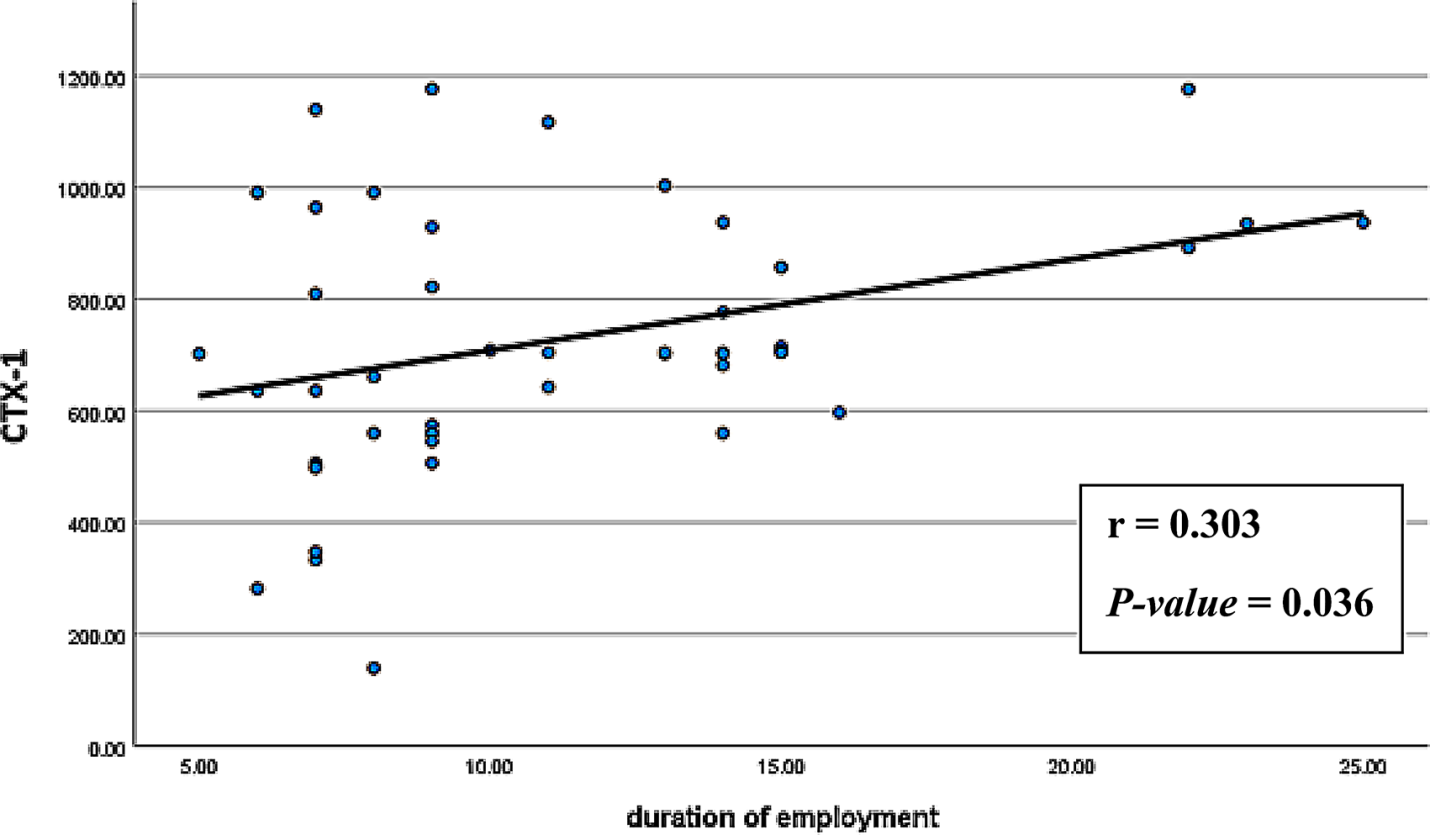
Correlation between the duration (years) of employment and the serum levels of type I collagen cross-linked C-telopeptide (CTX-1) among the exposed group (*r* = 0.303, *P*-value = 0.036)

**Fig. 4:**
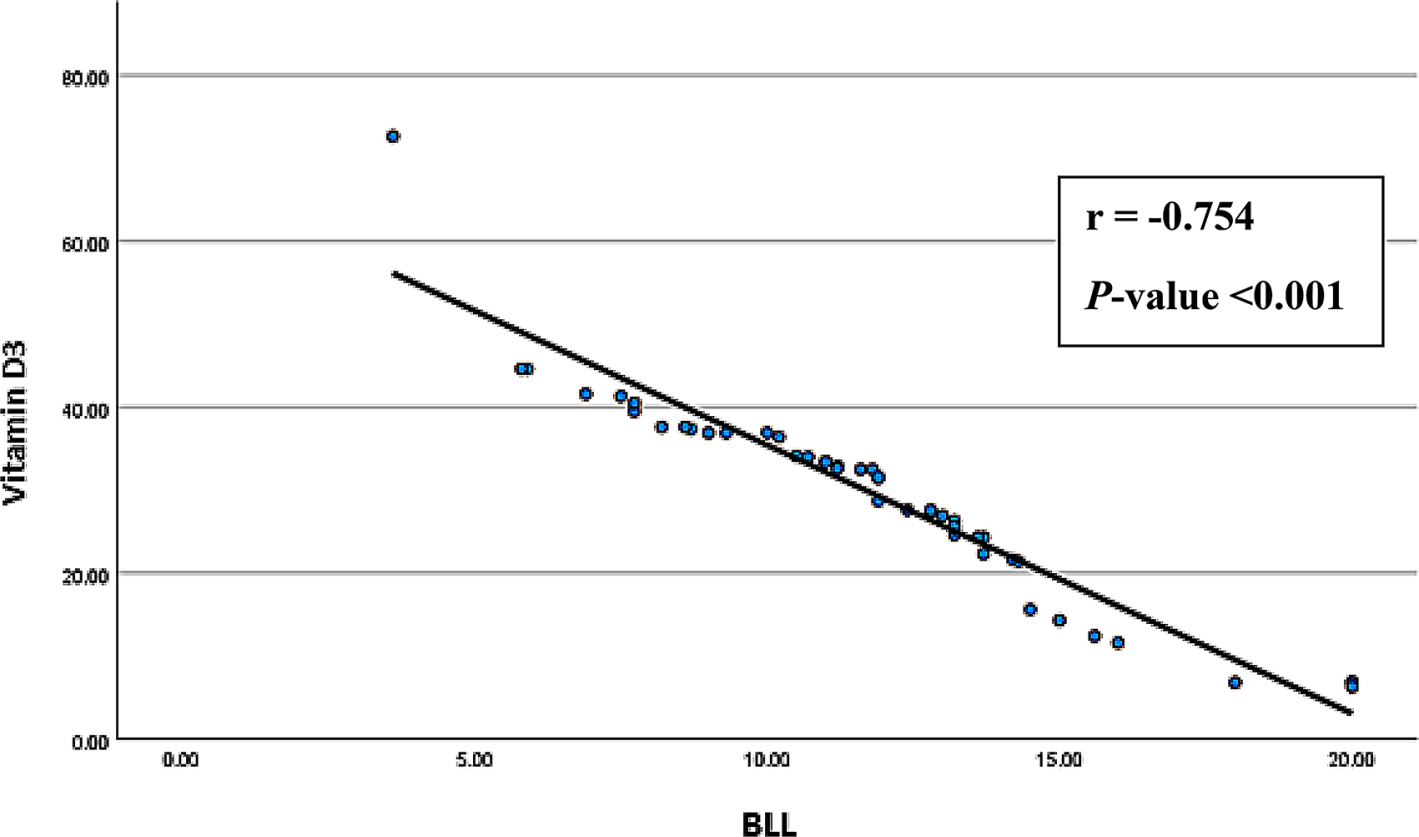
Correlation between the blood lead levels (BLL) and the serum levels of 1,25 dihydroxy-cholecalciferol (vitamin D3) among the exposed group (*r* = -0.754, *P*-value < 0.001)

**Fig. 5:**
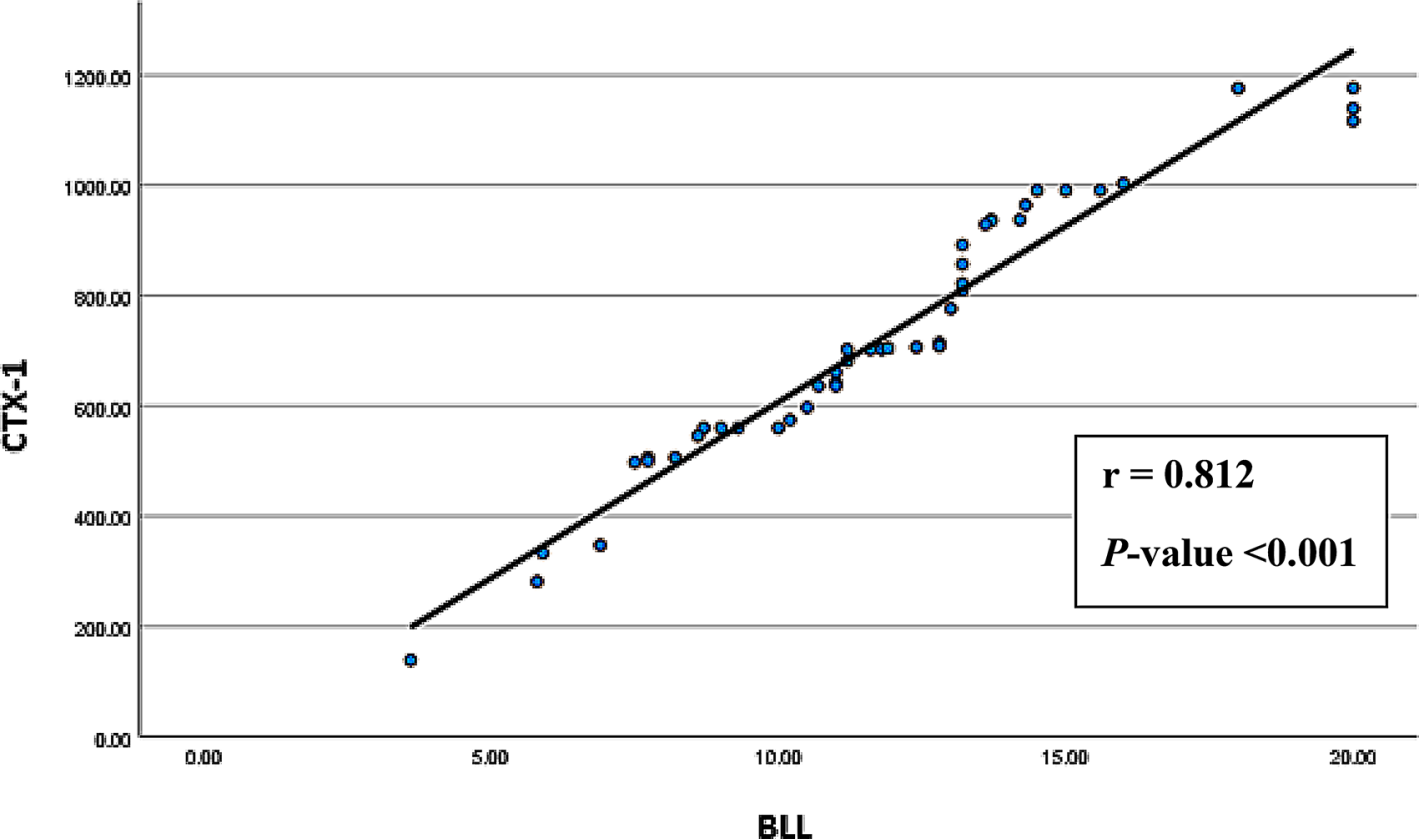
Correlation between the blood lead levels (BLL) and the serum levels of type I collagen cross-linked C-telopeptide (CTX-1) among the exposed group (*r* = 0.812, *P*-value < 0.001)

**Fig. 6:**
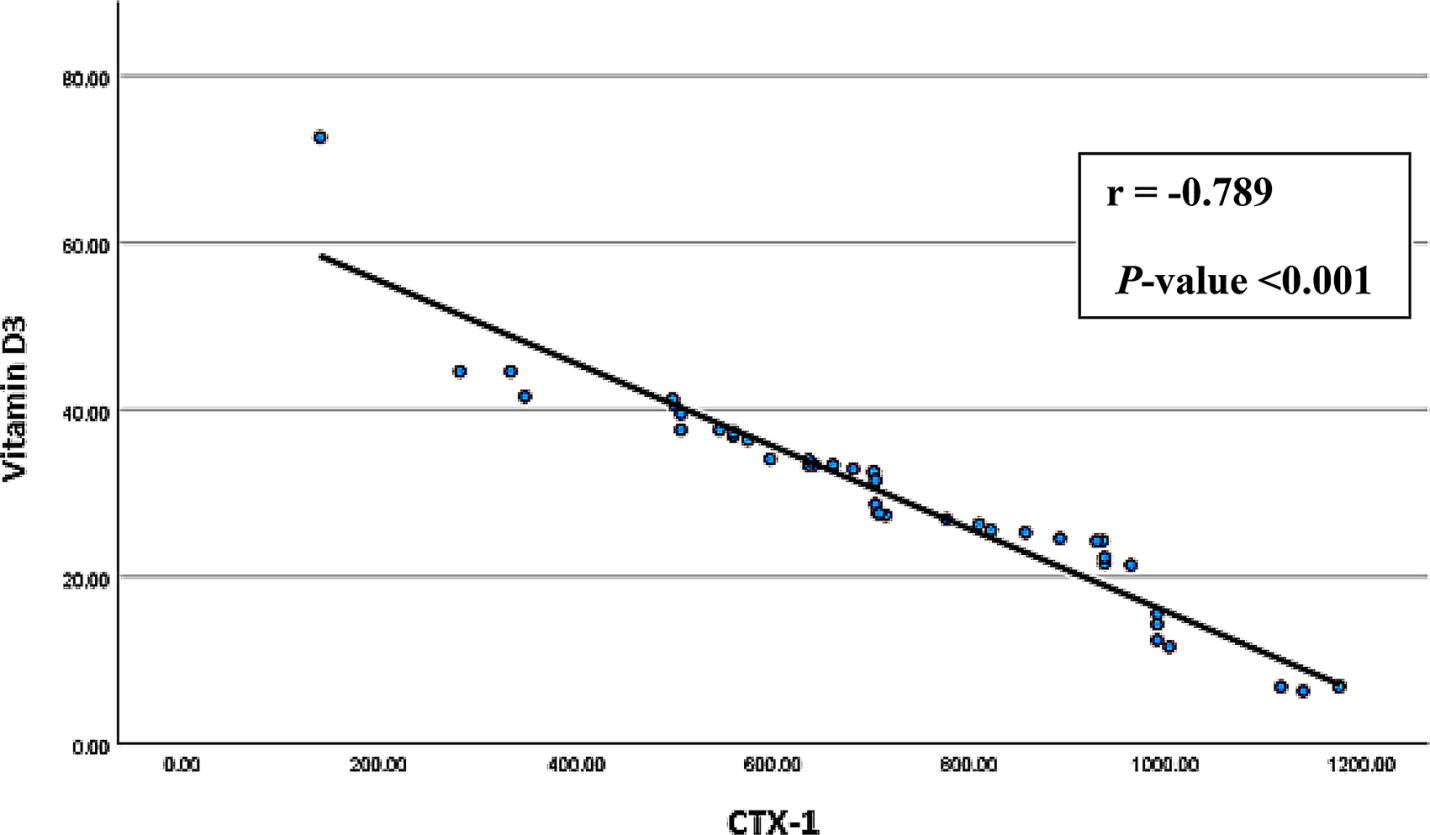
Correlation between the serum levels of type I collagen cross-linked C-telopeptide (CTX-1) and 1,25 dihydroxy-cholecalciferol (vitamin D3) among the exposed group (*r* = -0.789, *P*-value < 0.001)

Spearman’s correlation analyses were conducted to explore associations between age, BMI, smoking index, duration of employment, BLL, and serum levels of vitamin D3 and CTX-1 among exposed smelter workers. The results (Figs. 1, 2, 3, 4, 5 and 6) revealed significant positive correlations between the duration of employment and BLL (*r* = 0.289, *p* = 0.047), as well as between the duration of employment and serum CTX-1 levels (*r* = 0.303, *p* = 0.036).

A significant negative correlation was observed between the duration of employment and serum vitamin D3 levels (*r* = -0.297, *p* = 0.040). In addition, the results revealed a significant negative correlation between BLL and serum vitamin D3 levels (*r* = -0.754, *p* < 0.001), and a significant positive correlation between BLL and serum CTX-1 levels (*r* = 0.812, *p* < 0.001) among exposed participants. Furthermore, serum vitamin D3 levels showed a significant negative correlation with serum CTX-1 levels (*r* = -0.789, *p* < 0.001).

No significant correlations were identified between the three laboratory investigations (BLL, serum vitamin D3, and CTX-1) and the age, BMI, or smoking index of the exposed workers.

## Discussion

One of the most common heavy metals encountered during the smelting process is Pb, which is known to have harmful effects on human health. Previous research has shown that long-term exposure to Pb can disrupt bone tissue by either decreasing bone mineralization, through the inhibition of the kidney 1-α-hydroxylase enzyme (necessary for the synthesis of 1,25-dihydroxycholecalciferol, or vitamin D3), or by altering bone mass through the inhibition of chondrocytes and osteoblasts (Sharifi et al. 2011; Dongre et al. 2013; Akbal et al. 2014; Zhang et al. 2021). Pb may also accumulate in the bone cement, increasing the resorption of bone collagen. Collagen breakdown products, such as type I collagen cross-linked C-telopeptide (CTX-1), are used as biomarkers to indicate the presence of bone turnover (Ravibabu et al. 2020; Schini et al. 2023).

Focusing on the effect of occupational Pb exposure on workers’ bones in a non-ferrous smelter is justified because Pb has unique and well-documented pathways of deposition and toxicity in bone tissues. Unlike aluminium and copper, Pb has a high affinity for bone, accumulating over time and serving as a long-term marker of exposure. This storage affects bone remodeling, mineralization, and can lead to systemic effects when mobilized into the bloodstream. While exposure to other metals like aluminium may contribute to observed effects, their toxicokinetics and biological targets differ significantly from those of Pb. By isolating Pb’s impact on bones, researchers can more accurately assess its specific health risks and guide targeted interventions for Pb exposure, which remains a major occupational hazard in smelters. This approach ensures clarity in understanding Pb’s role despite the presence of other metals.

In the current study, the mean value of the blood lead levels (BLL) among the exposed smelter workers (11.86 ± 3.56 µg/dL) showed a statistically significant elevation compared to the comparison group (4.30 ± 1.40 µg/dL). Similarly, a recent Egyptian study showed that the mean BLL among the exposed workers in a copper smelter (27.81 ± 6.48 µg/dL) was significantly higher than among the administrative controls (10.67 ± 1.57 µg/dL) (Mourad and El-Sherif 2022). Also, in a previous Korean study, the BLL mean was significantly elevated among smelting workers (5.839 µg/dL) compared to the general male population (2.44 µg/dL) (An et al. 2017).

Notably, the World Health Organization (WHO) recommends a blood lead level of 5 µg/dL as the threshold for initiating a comprehensive examination of Pb exposure and taking action to minimize or eliminate it (WHO 2021). However, BLLs below 5 µg/dL have been associated with adverse health effects on multiple organ systems. The Centers for Disease Control and Prevention (CDC) has emphasized that no level of Pb exposure is entirely safe, particularly for vulnerable populations such as workers with prolonged exposure (CDC 2024b).

The smelter workers exposed to Pb in the present study showed statistically significant lower serum levels of vitamin D3 compared to the administrative controls. This is consistent with the findings of Batra and colleagues (2020) in India, who reported significantly decreased serum levels of vitamin D (28.82 ± 10.81 ng/ml) and significantly elevated blood lead levels (BLL) (38.02 ± 19.92 µg/dl) in Pb-exposed subjects compared to their controls. Similarly, Himani and colleagues (2020) observed high BLL and low serum vitamin D in workers in the battery industry in the Delhi NCR region, India. Additionally, similar results were found among workers exposed to Pb in the jewellery industry in Bangladesh (Mazumdar et al. 2017).

Bone is considered to be essential for organ protection, movement, and bodily support. Osteoclast-mediated bone resorption and osteoblast-mediated bone growth are interdependent. Biomarkers of bone turnover provide a means of quantifying the dynamic processes of bone production and resorption. In clinical research, the International Osteoporosis Foundation suggests utilizing C-telopeptide (CTX) as a measure of bone resorption (Schini et al. 2023). In the current study, compared to the control group, the exposed workers’ serum levels of CTX-1 showed a statistically significant rise. Based on a prior study, Pb exposure affects bone resorption, which can lead to osteopenia. According to the study, Pb may have a part in the genesis of osteoporosis and that the disease could alter bone turnover causing variations in Pb content in bones (Brito et al. 2014). In contrast to the control group, which consisted of office workers, a more recent study revealed statistically significant elevated levels of the collagen degradation product (hydroxyproline in urine, or HyP-U) among workers who were occupationally exposed to lead (Pb) in a Pb-battery manufacturing plant located in Tamil Nadu, India (Kalahasthi et al. 2021).

According to Patrick (2006), the review of literature revealed that the musculoskeletal system is susceptible to Pb poisoning, even at low levels of exposure. This can have an impact on bone growth and development, dentition, bone density, fracture healing, joint functioning, and motor abilities. In adults, more than 90% of the Pb body load is found in bone. In accordance to research on animals, Pb poisoning causes fibrous non-union and delayed fracture healing because of the acceleration of endochondral ossification (Carmouche et al. 2005). Medical history taking and clinical examination of the studied groups in the current study revealed a statistically significant elevated prevalence of several manifestations denoting skeletal system affection among exposed workers compared with their controls. The most commonly encountered complaints were recurrent generalized bony aches, prolonged low back pain, pain in multiple joints and a history of past fractures after minor traumas. Additionally, some exposed smelter workers reported limited joint movement while other workers gave a history of delayed healing of past fractures. Likewise, a recent study conducted in Tamil Nadu, India examined the musculoskeletal symptoms of long-term Pb exposure among employees of a Pb-battery manufacturing company. The findings revealed that among exposed individuals, lower back pain and discomfort were most common, followed by knee, shoulder, and neck pain. The study also found that even at low levels of Pb exposure, pain and discomfort in the neck, shoulders, and lower back were associated with signs of bone resorption (Kalahasthi et al. 2021). Furthermore, Ravibabu et al. (2019) identified a correlation between inflammatory markers and musculoskeletal diseases in workers exposed to lead (Pb) from Pb-battery production plants. Another study conducted in Tahran, Iran, came to the conclusion that fatigue and persistent bone aches could be related to elevated blood Pb levels (Aliasgharpour and Hagani 2005). In addition, those exposed to Pb experience severe fractures and heal more slowly than those who are not (Carmouche et al. 2005).

The logistic regression analysis in the current study pointed to the significance of the BLL as an independent predictor of bony aches and low back pain with an odds ratio greater than 1, denoting an increased risk of these aches with elevated BLL among the exposed workers. Moreover, the serum levels of vitamin D3 and CTX-1 were revealed to be significant predictors of low back pain and joint pain, respectively. Studies have shown a significant association between low serum vitamin D levels and the prevalence of chronic low back pain, emphasizing the need for adequate vitamin D intake for musculoskeletal health (Zadro et al. 2017; Alonso-Pérez et al. 2024). Also, earlier research had demonstrated a significant association between serum levels of CTX-1 and the severity of joint pain and tenderness (Olama et al. 2015).

The results of the correlation analysis in the present research revealed a significant positive correlation between BLL and the years of occupational exposure to Pb among the exposed participants. The same findings were observed in several previous studies (Rahimpoor et al. 2020; Al-Rudainy 2010; Mortada et al. 2001). While blood Pb is commonly used for assessing recent or current exposure due to its short half-life (about 30 days), it can still reflect accumulated Pb exposure over time, especially in workers who have been employed for extended periods. Long-term, low-level exposure can lead to Pb accumulation in soft tissues and bone, causing fluctuations in blood Pb levels even years after initial exposure. Thus, BLL can still be a valuable indicator of chronic exposure in settings where workers have consistently been exposed to Pb over several years, highlighting the importance of regular monitoring even in long-term exposure scenarios (Dang et al. 2024).

On the other hand, the significant negative correlation between decreased serum levels of vitamin D and elevated blood lead levels (BLL) due to prolonged Pb exposure has been confirmed by multiple earlier studies (Batra et al. 2020; Himani et al. 2020; Mazumdar et al. 2017). The significant positive association between elevated serum CTX-1 levels and years of occupational Pb exposure, as well as the positive correlation between serum CTX-1 levels and blood Pb, supports the idea of increased bone resorption with prolonged Pb exposure. Similar findings were reported by Kalahasthi and colleagues (2021), although they used a different collagen degradation product, urinary hydroxyproline. The significant association between serum levels of vitamin D3 and CTX-1 may also be explained by an additional mechanism beyond their association with BLL. When there is a deficiency of vitamin D, the body absorbs less calcium and produces more parathyroid hormone (PTH), which increases bone resorption and CTX-1 levels (Sarkissian et al. 2019). The linear regression analysis highlighted the associations of BLL, serum vitamin D3 levels, and CTX-1 among the exposed smelter workers. However, the duration (years) of Pb exposure was found to be a statistically significant independent predictor for serum CTX-1 levels only.

## Conclusions and recommendations

The study’s results suggest that high blood lead levels (BLLs) may have a significant impact on circulating vitamin D3 levels and bone resorption in workers occupationally exposed to Pb. The extrapolated data from this study could serve as a template to tailor guidelines for enhanced safety precautions and preventive actions aimed at improving working conditions and reducing worker exposure to Pb. The findings also emphasize the need for regular Pb exposure screening for employees. Those with BLLs exceeding the allowable limit should be informed about the harmful effects of Pb exposure and the importance of promptly implementing effective preventive measures. During routine physical examinations, serum levels of vitamin D3 and CTX-1 should be measured alongside BLL to detect any abnormalities. To maintain bone health and reduce musculoskeletal symptoms that may impair productivity, workers exposed to Pb should also consider taking supplements containing the active form of vitamin D.
